# Temperature-dependent expression of virulence genes in fish-pathogenic bacteria

**DOI:** 10.3389/fmicb.2015.00700

**Published:** 2015-07-09

**Authors:** José A. Guijarro, Desirée Cascales, Ana I. García-Torrico, Mario García-Domínguez, Jessica Méndez

**Keywords:** temperature, gene regulation, fish pathogen, bacterial virulence, aquaculture

## Abstract

Virulence gene expression in pathogenic bacteria is modulated by environmental parameters. A key factor in this expression is temperature. Its effect on virulence gene expression in bacteria infecting warm-blooded hosts is well documented. Transcription of virulence genes in these bacteria is induced upon a shift from low environmental to a higher host temperature (37°C). Interestingly, host temperatures usually correspond to the optimum for growth of these pathogenic bacteria. On the contrary, in ectothermic hosts such as fish, molluscs, and amphibians, infection processes generally occur at a temperature lower than that for the optimal growth of the bacteria. Therefore, regulation of virulence gene expression in response to temperature shift has to be modulated in a different way to that which is found in bacteria infecting warm-blooded hosts. The current understanding of virulence gene expression and its regulation in response to temperature in fish-pathogenic bacteria is limited, but constant extension of our knowledge base is essential to enable a rational approach to the problem of the bacterial fish diseases affecting the aquaculture industry. This is an interesting issue and progress needs to be made in order to diminish the economic losses caused by these diseases. The intention of this review is, for the first time, to compile the scattered results existing in the field in order to lay the groundwork for future research. This article is an overview of those relevant virulence genes that are expressed at temperatures lower than that for optimal bacterial growth in different fish-pathogenic bacteria as well as the principal mechanisms that could be involved in their regulation.

## Introduction

Bacteria are constantly subjected to different environmental influences, mainly related to their particular niche or lifestyle. One of the relevant factors influencing bacterial processes is temperature. Bacteria have to adapt their physiology to changes in temperature by adjusting their activities accordingly. Temperature accommodation is not only an acceleration–deceleration modulation of the whole cell’s enzymatic activity but also affects membrane-associated functions and leads to changes in bacterial gene expression (Schumann, 2012). In many cases, these changes are caused even by small temperature changes. Thus, bacteria have developed precise and defined regulation systems to modulate the expression of specific genes in response to moderate temperature shifts (Eriksson et al., 2002; Shivaji et al., 2010; Steinmann and Dersch, 2013). In addition, bacteria also have response mechanisms to drastic changes in temperature. The paradigms of this adaptation are the cold and heat shock response systems under which the bacteria induce a fast response to sudden and extreme temperature shift (Guisbert et al., 2008; Shamovsky and Nudler, 2008; Barria et al., 2013). In any case, temperature variations involve remodeling gene expression through different temperature sensor systems that recognize this environmental alteration and trigger an adequate response (Shivaji et al., 2010; Steinmann and Dersch, 2013).

In particular, temperature adaptation is essential during the infection process of endothermic organisms by pathogenic bacteria. These bacteria have to adjust their physiology to the host temperature, which is usually higher than that encountered within a vector or in the natural environment. This accommodation may involve the induction of both virulence and metabolic genes (Konkel and Tilly, 2000). Different systems govern the induction of virulence gene expression by temperature in planktonic mammal-pathogenic bacteria such as those belonging to *Yersinia*, *Salmonella*, *Shigella*, *Escherichia*, *Vibrio*, and *Listeria* genera. The presence in the bacteria of virulence factors is unnecessary during the planktonic state but essential for the infection process. Switching between environmental and host niches is sensed by the cell as a group of variations in different parameters including temperature. In this way, bacteria save energy by not expressing virulence genes until they sense they have entered the host environment.

While molecular mechanisms governing the expression of virulence factors in relation to temperature in bacteria pathogenic to mammals, and particularly to humans, have been studied in detail (see excellent review, Konkel and Tilly, 2000; Johansson and Cossart, 2003; Steinmann and Dersch, 2013), little is known about the temperature-regulated virulence factors in fish-associated bacterial pathogens and even less about the systems involved in their regulation.

**FIGURE 1:**
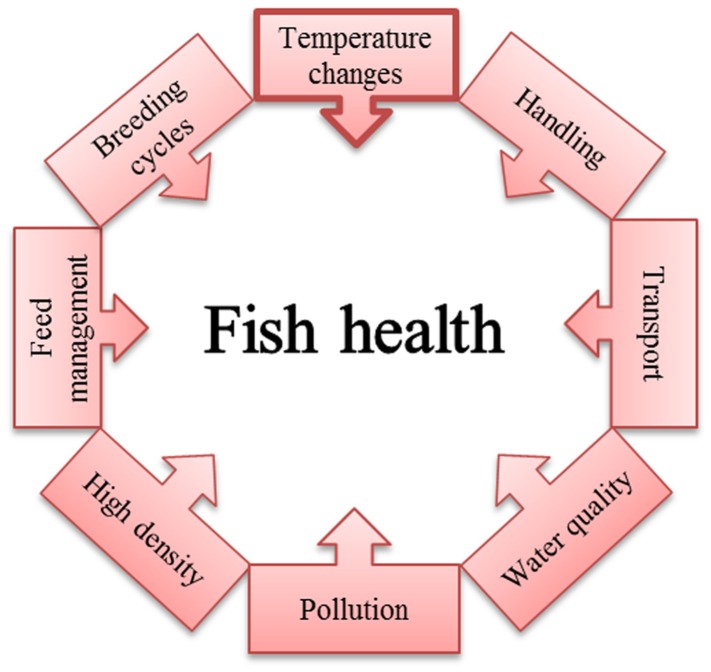
**Diagrammatic representation of the main factors affecting outbreaks of bacterial infectious diseases in the aquaculture industry.** Outbreaks are usually the result of changes in environmental temperature by itself or in association with some of the indicated stressing factors compromising fish health.

Disease development in fish is a complex process involving the interaction of a susceptible host, a virulent microorganism, and environmental factors. It is clear that in the aquaculture industry the third requirement is particularly important since the high densities and stress to which fish are commonly subjected favor the appearance of diseases that are infrequent or even non-existent in natural environments (**Figure 1**). A key environmental stress factor in outbreaks of most fish bacterial diseases in fish farms is water temperature. In some cases, outbreaks occur when water temperature drops to a certain value: Cold water vibriosis, (Enger et al., 1991); Cold water disease (Cipriano and Holt, 2005); Redmouth disease (Fernandez et al., 2007a). In others, such as: Lactococcosis (Vendrell et al., 2006), Haemorrhagic septicaemia (Austin and Austin, 2007), and Edwardsiellosis (Mohanty and Sahoo, 2007) outbreaks are related to an increase in water temperature. Interestingly, a remarkable number of bacterial diseases in aquaculture, particularly those of freshwater, occurred at temperatures below that of the optimal growth (TBO) of the infecting bacteria considering the optimum growth temperature for a particular bacterium to be that at which the fastest growth rate was observed under laboratory conditions. This temperature is usually higher than those found in the aquatic environment where the bacteria live or within their ectothermic hosts. Therefore, virulence gene expression in these bacteria should be regulated in such a way that maximum expression must occur at TBO. Nevertheless, since the body temperature of ectothermic animals is very close to the environmental temperature, additional factors must be necessary for full virulence, such as a co-regulation interaction between temperature and host metabolic processes.

## Sensing Temperature Changes in Mammal-Pathogenic Bacteria

Different molecular mechanisms are involved in sensing temperature changes in mammal-pathogenic bacteria (Konkel and Tilly, 2000; Johansson and Cossart, 2003; Han et al., 2013; Steinmann and Dersch, 2013). Most of them are related to changes in DNA, RNA or protein conformation and alteration in membrane structure (rigidification) which determines in some cases the activation of a two-component signal transduction pathway (Steinmann and Dersch, 2013). It should be pointed out that some of these thermo-sensing systems could not work in fish-pathogenic bacteria. One example is the H-NS-mediated repression of virulence gene expression by binding to AT-DNA regions at low temperature. In this case, induction of virulence genes depends on an increase in temperature, corresponding to the entry into the host (37°C), which causes H-NS protein to be released from the AT-DNA regions, thus derepressing transcription and also enabling binding of transcriptional activators and so allowing expression of the regulated genes (Dorman, 1996; Hurme and Rhen, 1998; White-Ziegler and Davis, 2009). The H-NS protein is a universal regulator of the bacterial genome, particularly relevant in *Enterobacteriaceae*. Examples of its action are the regulation of the *virF*, *ssrB*, and *ymoA* genes, involved in the virulence of *Shigella flexneri* (Falconi et al., 1998), *Salmonella enterica* (Fass and Groisman, 2009), and *Yersinia species* (Bohme et al., 2012), respectively. In fish-pathogenic bacteria, it is difficult to imagine how this system could work, since the temperature encountered by bacteria within the host is generally lower than their optimum growth temperature, this being the opposite of what happens in mammal-pathogenic bacteria.

**FIGURE 2:**
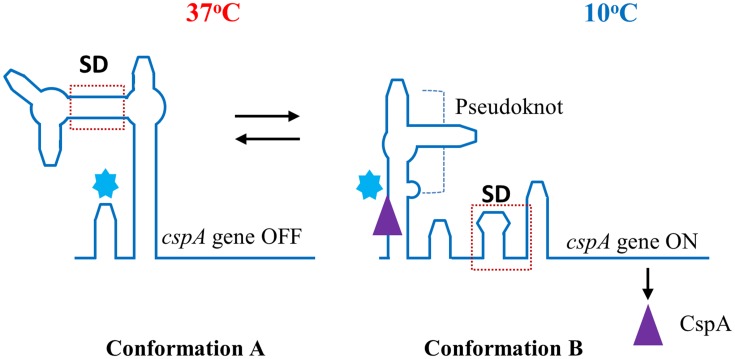
**Transcription of the gene encoding the cold shock protein CspA depends on an mRNA rearrangement at low temperature. (A)** Interaction of the 5′ untranslated region and the coding region blocks the Shine–Dalgarno (SD) sequence and represses translation of *cspA* mRNA at 37°C. **(B)** A cold shock (10°C) produces the liberation of the SD sequence which allows expression of *cspA* gene. Conformation of the mRNA at 10°C is stabilized by pseudoknot formation. This is further abolished after binding of CspA (▲) to the cold box (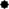) in the late phase of cold shock adaption.

RNA thermometers modulate translation efficiency of a particular mRNA in relation to temperature (Eriksson et al., 2002; Johansson and Cossart, 2003; Kortmann and Narberhaus, 2012; Han et al., 2013; Steinmann and Dersch, 2013; Grosso-Becerra et al., 2014; Weber et al., 2014). They are sequences able to form intramolecular stem-loop structures affecting the ribosomal binding site (RBS) and the translation initiation codon. In that way, at low temperature, the mRNA conformation makes the RBS site inaccessible to the ribosome. When temperature increases and, in particular, at host temperature (37°C), there is a stem-loop melting with a conformational change at the mRNA 5′end, rendering the RBS accessible to the ribosome and making mRNA translation possible. This system depends on the high stability at low temperatures of mRNA 5′sequences involved in sequestering RBS. Therefore, it seems that this mechanism would not be appropriate for regulating virulence gene expression at TBO in fish-pathogenic bacteria. However, an RNA thermo-switch has already proved to be involved in gene regulation at TBO and it could be a system implicated in the regulation of virulence genes in fish-pathogenic bacteria. Thus, at optimal bacterial growth temperatures, the RNA forms stem-loops sequestering RBS and preventing virulence gene expression, whereas at TBO, RNA conformation changes, resulting in an accessible RBS and the initiation of translation (Kortmann and Narberhaus, 2012; Steinmann and Dersch, 2013). An example of this kind of regulation system is the *cspA* gene of *Escherichia coli* involved in the cold shock response (Yamanaka et al., 1999; Giuliodori et al., 2010). Indeed, *cspA* mRNA undergoes a structural rearrangement at low temperature in relation to the conformation at 37°C, resulting in more efficient translation. At 37°C the 5′end of the transcribed *cspA* mRNA forms a secondary structure in which RBS is occluded, whereas at 10°C, an entirely new secondary structure is formed, leaving the RBS sequence accessible to the ribosome (Giuliodori et al., 2010; **Figure 2**).

In addition, regulation by trans-acting non-coding RNAs, which has been studied in other pathogens, might be an interesting area to explore in fish-pathogenic bacteria. This basically involves the temperature regulation of *rpoS* expression, a general stress response sigma factor in bacteria. The effects of RpoS on pathogenesis are highly variable and depend on the species (Dong and Schelhorn, 2010). Nevertheless, it is well established that induction of RpoS by small non-coding RNAs is enhanced during growth at low temperatures (Rapoila and Gottesman, 2001; Rapoila et al., 2003; Lybecker and Samuels, 2007). This process is dependent on the presence of the sRNA DsrA (Kortmann and Narberhaus, 2012; Han et al., 2013). Expression of DsrA is enhanced at low temperature, resulting in base pairing with *rpoS* mRNA in the 5′ non-co ding region, bringing about an increase in *rpoS* translation (Rapoila and Gottesman, 2001; Rapoila et al., 2003; McCullen et al., 2010). Thus an RpoS-like system could be one of the mechanisms able to activate virulence factors in fish-pathogenic bacteria at TBO.

**FIGURE 3:**
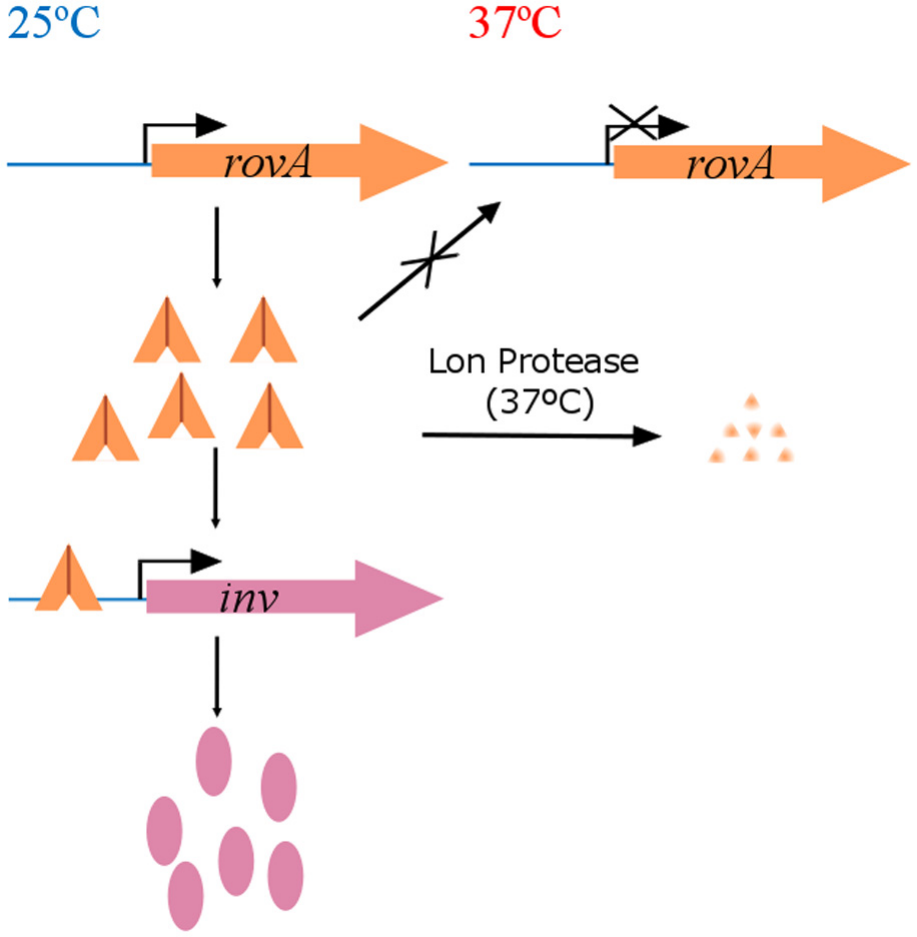
**Thermoregulation of the invasin gene in *Yersinia* species through RovA.** RovA is a transcriptional regulator able to bind at TBO in a dimer conformation to the promoter region of the invasin gene (*inv*) activating its expression. *Inv* is involved in the first steps of the infection process in human pathogenic *Yersinia*. When temperature reaches 37°C this dimer undergoes a conformational change that makes unable its binding to the DNA. In this condition, expression of the *inv* gene does not occur and RovA is degraded by the Lon protease.

Temperature-dependent gene expression could also be mediated by means of proteins. Amongst the different regulation mechanisms in which proteins are involved, those based on repressing promoter activity at TBO by protein DNA binding and further protein-DNA disassembling at host temperatures (37°C) deserve special attention. Examples of this kind of regulation are the TlpA and HtrA proteins of *S. enterica* (Gal-Mor et al., 2006) and in *Helicobacter pylori* (Hoy et al., 2012), respectively, both involved in the virulence of these bacteria. These regulation systems are of interest, but, may not participate in virulence gene modulation in fish-pathogenic bacteria. However, it is important to consider two other mechanisms that could well be involved in virulence gene regulation at TBO: protein conformational changes that abolish DNA-binding at host temperature (37°C), stopping gene transcription, i.e., the RovA system in *Yersinia* species (Ellison et al., 2004; Marceau, 2005; Cathelyn et al., 2006); and the repressor/antirepressor complex MogR:GmaR regulating motility of *Listeria monocytogenes* (Kamp and Higgins, 2011). In both cases, gene expression takes place at TBO and it is impaired at host temperature (37°C). In *Yersinia enterocolitica*, RovA binds at 25°C at the 5′end of the *inv* gene, activating its transcription to produce invasin, a protein involved in the first steps of tissue colonization (Ellison et al., 2004; **Figure 3**). At 37°C a conformational RovA change prevents its binding to DNA and makes it susceptible to degradation by the Lon protease (Herbst et al., 2009), impeding gene expression (Cathelyn et al., 2006; Ellison and Miller, 2006; **Figure 3**). As in *Yersinia*, the first steps of the infection process in *L. monocytogenes* imply the activation of genes at temperatures below 30°C. In particular, genes related to motility are needed for bacterial entry into the host cells (O’Neil and Marquis, 2006). This flagelar motility (*flaA* gene) is temperature-regulated through the GmaR:MogR complex, which once bound to the upstream promoter region, enables *flaA* gene expression of *L. monocytogenes* at 30°C (Kamp and Higgins, 2011). Conformational changes in GmaR at 37°C prevent its union to MogR, which is thus able to act as a repressor of the *flaA* gene by itself (Shen and Higgins, 2006), blocking its expression and rendering the bacterial cells non-motile just after invasion has occurred.

## Sensing Temperature Changes in Plant-Pathogenic Bacteria

Thermoregulated expression of virulence factors in plant-associated bacteria was first summarized by Smirnova et al. (2001). So far, two-component systems are the only ones described as thermosensing mechanisms related to virulence gene expression at TBO in plant-pathogenic bacteria. *Agrobacterium tumefaciens* VirAG (Jin et al., 1993) and *Pseudomonas syringae* CorSR are both involved in tumor formation and virulence-enhancing phytotoxin production, respectively. In *A. tumefaciens*, the etiological agent of “Crown Gall disease” in plants, low temperatures play a relevant role in the induction of virulence gene expression. This induction is in part due to the VirAG two-component regulatory system in which VirA undergoes a reversible conformational change at temperatures greater than 32°C that inactivates the sensor kinase (Melchers et al., 1989; Jin et al., 1993). So, VirA constitutes a thermosensor related to the progression of the infection processes at temperatures below 32°C. In the same way, the bacterial blight pathogen *P. syringae* produces the phytotoxin coronatine in a temperature-dependent manner (Palmer and Bender, 1993; Ullrich et al., 1995; Arvizu-Gómez et al., 2013). The regulatory mechanism involved in the synthesis of this toxin is the CorRS two-component system in which the histidine kinase autophosphorylation of the histidine kinase CorS is abolished at 28°C, whereas at 18°C this membrane protein has the phosphorylation domain accessible (Palmer and Bender, 1993; Ullrich et al., 1995; Arvizu-Gómez et al., 2013). Therefore, at the appropriate temperature (18°C), CorS phosphorylation triggers the expression of the biosynthetic operons encoding coronatine.

## Temperature-Dependent Virulence Gene Expression in Fish-Pathogenic Bacteria

Different fish-pathogenic bacteria produce diseases in their host in response to TBO, which seems to be a key element for these bacteria to produce virulence factors. This is not exclusive to fish pathogens but also true for infection in plants (Smirnova et al., 2001), and probably in molluscs (Romalde et al., 2014). Depending on the niche of these bacteria, an additional characteristic should be considered in gene temperature regulation. In fact, the environmental temperature encountered by these bacteria is, in many cases, similar to that found in the ectothermic host. Therefore, it may be deduced that in order to prevent energy waste it is probable that additional factors, other than temperature, must be frequently involved in virulence gene regulation during host invasion and colonization. Otherwise, virulence genes in these bacteria would be on in the planktonic state at environmental temperature.

As described above, some of the systems regulating temperature-dependent virulence factor expression in bacteria pathogenic to mammals could not function in fish- pathogenic bacteria because a large number of these pathogens induce virulence factors at TBO.

Different approaches have been undertaken in order to identify up-regulated virulence genes at TBO in fish-pathogen bacteria.

## Identification of Virulence Genes Induced at TBO

### Yersinia ruckeri

*In vivo expression technology* (IVET) was used for the selection of specifically *in vivo* induced genes in *Y. ruckeri*, the etiological agent of the “Enteric red mouth disease” of salmonids, (Fernandez et al., 2004). Expression of some of the genes selected was found to be higher at 18°C, the temperature at which outbreaks of disease occur, than at 28°C, the optimal bacterial growth temperature. Thus, the expression of the *traHIJKCLMN* operon encoding a putative type IV secretion system involved in the virulence of the bacterium was reduced by 64% upon incubation at optimal growth temperature in relation to 18°C (Méndez et al., 2009; **Table 1**).

**Table 1:** Virulence-related genes which are up-regulated at TBO in fish-pathogenic bacteria.

Pathogen	Disease	Virulence related factor up-regulated at TBO	Function	Reference
*Yersinia ruckeri*	Redmouth disease	*rucC-rupDGC*	Ruckerbactin (siderophore production)	Fernandez et al. (2004)
		*yrp1*	Metalloprotease	Fernandez et al. (2002, 2003)
		*yhlA*	Haemolysin	Fernandez et al. (2007b)
		*traH-N*	Type IV SS	Méndez et al. (2009)
				
*Flavobacterium psychrophilum*	Cold water disease	FP1516	Two component histidine kinase	Hesami et al. (2011)
		FP0666	ATP-dependent RNA helicase	Hesami et al. (2011)
		FP0834	ATP-binding cassette transporter	Hesami et al. (2011)
		FP1619	Metalloprotease (M43 Cytophagalysin family)	Hesami et al. (2011)
		FP2096	Outer membrane protein	Hesami et al. (2011)
				
*Lactococcus garvieae*	Lactococcosis	*rpoE*	Delta subunit RNAp	Aguado-Urda et al. (2013)
		*potABCD* operon	Cold response	Aguado-Urda et al. (2013)
		gene 25, gene 51, gene 20	Autolytic enzymes	Aguado-Urda et al. (2013)
				
*Aeromonas hydrophila*	Haemorrhagic septicaemia	–	Serin metalloprotease	Yu et al. (2007)
		–	*S*-layer	Yu et al. (2007)
		–	Flagellins	Yu et al. (2007)
		–	T3SS	Yu et al. (2007)
		–	Outer membrane components	Yu et al. (2007)
				
*Edwardsiella tarda*	Edwardsiellosis	*phoP–phoQ*	Two component system	Srinivasa Rao et al. (2004), Zheng et al. (2005), Chakraborty et al. (2010)
		*eseBCD*	T3SS	Srinivasa Rao et al. (2004), Zheng et al. (2005), Chakraborty et al. (2010)
		*evpA-H*	T6SS	Srinivasa Rao et al. (2004), Zheng et al. (2005), Chakraborty et al. (2010)
		*sip1*	Zinc metalloprotease	Lv et al. (2013), Zhou et al. (2015)
				
*Aliivibrio salmonicida*	Cold water vibriosis	*litR*	Two component system	Bjelland et al. (2012), Hansen et al. (2014)
		–	Siderophore production	Colquhoum and Sorum (2011)
		*luxI/luxR*	Quorum sensing	Hansen et al. (2015)
		*ainS/ainR*	Quorum sensing	Hansen et al. (2015)
				

In the same way, the expression of two genes encoding extracellular proteins involved in bacterial virulence were also temperature regulated. The expression of YhlA haemolysin and Yrp1 protease was approximately seven and threefold higher at 18°C than at 28°C, respectively (Fernandez et al., 2002, 2003, 2007b). *In vivo* expression of Yrp1 in rainbow trout kept at 18°C was confirmed by using the *lux* operon as a reporter system (Méndez and Guijarro, 2013). Ruckerbactin, a cathecholate siderophore iron acquisition system was also regulated by temperature (Fernandez et al., 2004; **Table 1**). Therefore, the temperature-dependent modulation of virulence genes in *Y. ruckeri* tends to optimize the expression of these in conditions mimicking those encountered in the host. However, despite the important work carried out in the regulation of virulence genes in human pathogenic *Yersinia* species, there is no study related to how *Y. ruckeri* regulates virulence gene expression at TBO. It seems that some of the temperature sensing systems existing in human pathogenic Yersinia species such as H-NS and conformational changes in the structure of the 5′end in mRNA would be unlikely to work, at least in a similar way, in *Y. ruckeri* at TBO. However, RovA, RpoS as well as two-component systems could be relevant in the *Y. ruckeri* virulence expression at TBO.

### Flavobacterium psychrophilum

The Gram-negative bacterium *F. psychrophilum* is the etiological agent of “Bacterial cold water disease,” one of the major causes of economic losses in the salmonid aquaculture industry. The disease occurs at temperatures below 14°C, 20°C being the optimal growth temperature of the bacterium (Cipriano and Holt, 2005; Starliper, 2011). Therefore, it is clear that expression of virulence genes at TBO is a key element in the infection process. The first indication of this came from the studies related to the extracellular metalloproteases Fpp1 and Fpp2 (Secades et al., 2001; Pérez-Pascual et al., 2011; Gómez et al., 2012). Extracellular proteolytic activity was linked by different authors to the virulence of this bacterium due to its potential role in the degradation of host tissues (Bertolini et al., 1994; Ostland et al., 2000). Interestingly, Fpp1 and Fpp2 metalloproteases of *F. psychrophilum* are overproduced at 12°C in relation to 18°C (Secades et al., 2001; Gómez et al., 2012). This up-regulated gene expression at 12°C was confirmed by transcriptional fusion using *gfp* as a reporter gene (Gómez et al., 2012).

TBO-regulated genes in this bacterium were identified by Hesami et al., (2011). Using suppression subtractive hybridization (SSH) a set of genes that were up-regulated at 8°C versus 20°C were defined (**Table 1**). Among them should be highlighted a histidine kinase temperature sensor belonging to a two-component system. This sensor is similar to the LytS involved in the regulation of cell autolysis (Brunskill and Bayles, 1996) and biofilm formation in different bacteria (Sharma-Kuinkel et al., 2009), and whose expression increased about 18-fold at 8°C versus 20°C. Another gene that was identified encodes an ATP-dependent RNA helicase, which is up-regulated approximately 11-fold at 8°C and whose function could be to facilitate the initiation of transcription at low temperature by destabilizing the mRNA secondary structure (Schmid and Linder, 1992; Lim et al., 2000). In addition, this kind of enzyme has been involved in the regulation of bacterial virulence in *H. pylori* and *Clostridium perfringens* (Heung and Del Poeta, 2005). As was described above, extracellular proteolytic activity is related to pathogenesis. In particular, zinc-dependent metalloprotease activity was suggested to play an important role in muscle necrosis in rainbow trout with *F. psychrophilum* infection (Ostland et al., 2000). In the SSH study, the M43 cytophagalysin zinc-dependent metalloprotease gene was induced at 8°C, suggesting its involvement in the infection process; DNA gyrase subunits A and B were also induced at 8°C. GyrA had already been described as a cold-induced protein in bacteria (Scherer and Neuhaus, 2006) and its role seems to be related to the increase in DNA negative supercoiling at TBO. Additionally, an ABC transport system, an outer membrane protein antigen and a recombinase (recA), as well as four housekeeping genes were also up-regulated at 8°C (Hesami et al., 2011). All the SSH-identified genes were up-regulated in 12 different strains of *F. psychrophilum* from different origins, indicating that the induction of these genes at TBO is a common process in the species.

### Lactococcus garvieae

*Lactococcus garvieae* is a ubiquitous and widely distributed Gram-positive bacterium. It is the causative agent of the fish disease “Lactococcosis” (Vendrell et al., 2006), although it can also produce septicaemia in humans as an opportunistic pathogen (Russo et al., 2012). Lactococcosis is one of the most relevant diseases affecting farmed fish species, particularly rainbow trout (*Oncorhynchus mykiss*; Vendrell et al., 2006; Reimundo et al., 2011) and outbreaks occur at water temperatures around 18°C. The bacterium has also been isolated from different animal sources, including cows, pigs, cats, and horses (Aguado-Urda et al., 2010) and even from foods, meat and dairy products (Ferrario et al., 2012). The ability to grow in such different environments and to infect both endothermic and ectothermic animals, suggests the existence of changes in gene expression, and in particular, changes affecting virulence genes. The expression of these depends on the environmental signals encountered inside the host and so the temperature range over which *L. garvieae* expresses virulence factors could be really wide, varying from 18 to 37°C.

An interesting study using transcriptome analysis was assessed in two *L. garvieae* strains isolated from fish and humans to investigate the effect of growth temperature (18°C vs. 37°C) on differential gene expression (Aguado-Urda et al., 2013). Interestingly, in the fish-pathogenic strain, several genes linked to virulence were up-regulated at 18°C vs. 37°C (**Table 1**). This was the case of *rpoE*, encoding the delta subunit of RNA polymerase. In addition to its role in global regulation during environmental adaptation, this gene has been linked to virulence in Gram-positive and Gram-negative bacteria such as *Streptococcus agalactiae* (Jones et al., 2003; Seepersaud et al., 2006) and *Vibrio harveyi* (Rattanama et al., 2012), respectively. Three genes related to virulence in gram-positive bacteria and involved in autolysis have also been identified. The role of these genes in pathogenesis seems to be related to the release of membrane and wall compounds during bacteriolysis, which would act on macrophages and would induce an immune response resulting in septic shock (Ginsburg, 2002). Finally, other genes over-expressed at 18°C were those belonging to the cold response polyamine transport operon *potABCD*, which is similar to that involved in the pathogenesis of *S. pneumoniae* and necessary for survival of the bacteria in host environments (Ware et al., 2006).

### Aeromonas hydrophila

*Aeromonas hydrophila*, a ubiquitous Gram-negative bacterium, is an opportunistic pathogen of different endothermic animals, including humans, as well as ectothermic hosts such as rainbow trout (Thune et al., 1993; Austin and Austin, 2007). It can grow at temperatures ranging from 4 to 42°C (Rouf and Rigney, 1971), although the capacity to grow at the extremes of this range varies among strains. Different studies have clearly established that TBO is an inductor of virulence gene expression in *A. hydrophila*. The composition of the extracellular proteome produced at 25 versus 37°C showed that the total amount of extracellular products was significantly lower at 37°C than that at 25°C in spite of the growth rate being greater at 37°C (Yu et al., 2007). A Maldi-Toff analysis of these extracellular products showed a higher production at 25°C of a serin-metalloprotease, *S*-layer and flagellins among others, than was seen at 37°C (Yu et al., 2007; **Table 1**). In the same way, proteins related to the type III secretion system were also up-regulated at 25°C (Yu et al., 2007). TBO also had an influence on the composition of outer membrane components and the virulence of this pathogen (Yu et al., 2007). In fact, cells grown at 20°C showed higher levels of some phospholipid and different LPS aspect in relation to those cultured at 37°C (Yu et al., 2007). These results were further confirmed by the effect of the Wzz protein in the regulation of LPS chain length (Jimenez et al., 2008). The gene encoding Wzz showed a substantially greater level of expression at 20°C than that at 37°C, which resulted in higher LPS production at TBO with effects on the virulence of *A. hydrophila* (Merino et al., 1992). All of this, together with the fact that strains grown at 20°C were more virulent for fish and also for mice, indicates that TBO plays an essential role in the control of *A. hydrophila* virulence.

## Thermosensing Systems Involved in Virulence

### Edwardsiella tarda

**FIGURE 4:**
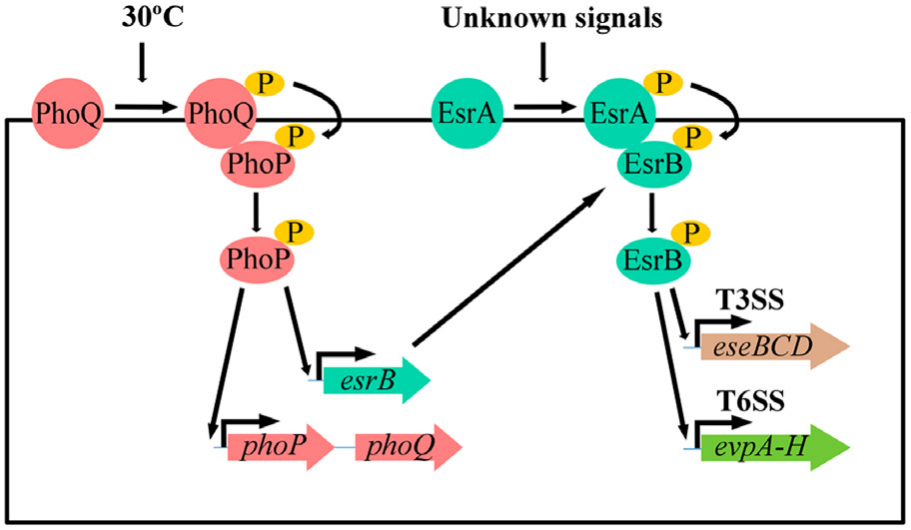
**Model for temperature signal transduction in *Edwardsiella tarda* by the PhoP–PhoQ two-component regulatory system.** PhoQ displays a different structure at 37°C as compared with 30°C. Autophosphorylation of PhoQ only takes place in a defined range of temperature around 30°C and leads to a subsequent phosphorylation of the response regulator PhoP. PhoP-P is now able to bind to upstream regions of *phoP–phoQ* and *esrB*, a response regulator of the EsrA–EsrB two component system, leading to an increase in the synthesis of both PhoP–PhoQ and EsrB. EsrB could be now phosphorylated by EsrA-P, a process depending on an unknown signal (s). EsrB-P binds to the promoter region of both virulence-related T3SS and T6SS activating their transcription.

*Edwardsiella tarda* infects many species of farmed fish, causing “Edwardsiellosis,” a haemorrhagic septicaemia that leads to important losses in aquaculture (Thune et al., 1993; Austin and Austin, 2007). This Gram-negative bacterium has a broad host range and also causes infections in higher animals, including humans, in which it causes gastrointestinal disorders (Plumb, 1993) and bacteraemia (Yang and Wang, 1999) amongst other pathologies (Osiri et al., 1997; Slaven et al., 2001). Although pathogenesis of *E. tarda* is multifactorial, the two-component system PhoP–PhoQ detects changes in environmental temperature (Chakraborty et al., 2010). Indeed, PhoQ is a histidine kinase which senses temperature changes through conformational modification in its secondary structures (**Figure 4**). As a result, autophosphorylation of PhoQ only takes places over a defined range of temperature around 30°C (**Figure 4**). This allows the transfer of the phosphate group from PhoQ to PhoP. When phosphorylated, PhoP binds to the promoter region of *esrB* and activates its transcription (**Figure 4**). EsrB is a response regulator of another two-component system (EsrA–EsrB). Phosphorylated EsrB binds to the promoter region of at least two clusters of genes codifying type III (EseBCD) and type VI (EvpA-H) secretion systems, activating their transcription (Srinivasa Rao et al., 2004; Chakraborty et al., 2010; **Figure 4**). Both Type III and Type VI secretion systems are associated with virulence in this bacterium (Srinivasa Rao et al., 2003, 2004; Zheng et al., 2005; Wang et al., 2009). Interestingly, expression of these clusters together with *esrB* was temperature-dependent and was higher at 25°C than at 37°C (Srinivasa Rao et al., 2004). In the same way, expression of *evpA* and *evpC* was reduced by 84% at 37°C when compared with expression at 25°C (Srinivasa Rao et al., 2004). Therefore, these genes, essential for virulence in *E. tarda*, were suppressed at 37°C. Besides, a total of 13 proteins in *E. tarda* were found to require the presence of PhoP for full expression, specifically the zinc metalloprotease Sip1 (Lv et al., 2013). This was found to be essential for serum resistance and host infection (Zhou et al., 2015), corroborating once more the relation between TBO induction of PhoP and *E. tarda* virulence. Protein secretion was also significantly lowered at 37°C in *E. tarda* compared to 25°C. In addition, in a challenge experiment, 90% of the fish injected with cells grown at 37°C survived, whereas 70% of the fish died when they received bacteria grown at 25°C (Srinivasa Rao et al., 2004). These results clearly established that the expression of these two protein secretion systems involved in the virulence of *E. tarda* was significantly lower at 37°C than at 25°C and depends on the PhoP–PhoQ system.

### Vibrio (Aliivibrio) salmonicida

*Vibrio salmonicida* causes “Cold water vibriosis” in farmed salmonid fish, a systemic disease with hemorrhages and anemia as the main signs (Holm et al., 1985; Egidius et al., 1986). Outbreaks of the disease appear in seawater and generally at temperatures below 10°C, although the species is able to grow until 20°C (Enger et al., 1991).

Quorum sensing is a well-defined cell-density-dependent regulation system in bacteria that is involved in the coordination of different activities related to pathogenesis. In *V. salmonicida* this quorum sensing system regulates biofilm formation through LitR (Bjelland et al., 2012; Hansen et al., 2014). Mutation of *litR* leads to greater adhesion, cell aggregation, biofilm formation, and higher motility in relation to the parental strain (Bjelland et al., 2012). Interestingly, the *litR* regulatory effects are under temperature regulation. At low temperature (4–8°C) there were important differences in biofilm formation and colony morphology between parental and litR mutant strains. Nevertheless, at temperatures above 10–12°C, the behavior of these strains was more similar to each other in relation to these phenotypes (Hansen et al., 2014). This shows that at temperatures close to that of the disease development, the negative regulatory effect of *litR* is weak or absent. Indeed, challenge experiments in Atlantic salmon using parental, *litR* mutant and *litR* complemented *V. salmonicida* strains showed a reduced mortality of the mutant in relation to the parental and complemented strains (Bjelland et al., 2012). All of this serves to indicate that temperature in *V. salmonicida* is a key factor involved in virulence regulation. Besides, LuxI/LuxR and AinS/AinR quorum sensing systems in *A. salmonicida* were recently shown to be dependent on growth temperature (Hansen et al., 2015). Indeed, *N*-acyl-homoserine lactones were efficiently produced by both systems when bacteria were grown at 6 or 12°C, whereas at 16°C this production decreased to values less than 5% of the maximum concentration found at 6°C (Hansen et al., 2015). Interestingly, LitR was found to be a positive regulator of both *luxI* and *ainS*. Therefore, a new link was established between maximum expression of quorum sensing systems at low temperature and virulence.

This is also supported by the effect of temperature on siderophore production and the regulation of iron outer membrane proteins in this bacterium (Colquhoum and Sorum, 2011). Hydroxamate siderophore was produced only at 10°C or less, and in the same way iron-regulated membrane proteins were suppressed at 15°C compared to the expression at 10°C or less (Colquhoum and Sorum, 2011; **Table 1**). Since iron chelator systems are usually virulence factors, it could be deduced that TBO is involved in the control of pathogenicity of this bacterium.

## Conclusion and Perspectives

From these limited studies that form the baseline to initiate further approaches in the field, it seems that extracellular enzymes, iron sequestering systems, bacteriolysis-related proteins, as well as secretion systems are some of the virulence factors which are up-regulated at TBO in fish-pathogenic bacteria. Two temperature-dependent regulatory systems have been described in fish pathogens: a two-component regulatory system in *E. tarda* working in a similar way to the ones described in mammals and plant pathogenic bacteria, and the *litR*, a negative regulator of *V. salmonicida* involved in blocking virulence-related genes expression.

A priori, only some of the existing regulatory systems in human pathogenic bacteria could be functional in fish-pathogenic bacteria (i.e., RovS, RpoS-like systems and different kinds of RNA thermometers). It is more than likely that new virulence regulation mechanisms are still to be discovered in this kind of bacteria. It is particularly noteworthy that in many cases there are no differences between the environmental and the host temperature. Therefore, if virulence genes in fish-pathogenic bacteria are induced exclusively under TBO, they should be active in the planktonic state. It is tempting to speculate that bacteria could have additional intertwined systems regulating the expression of virulence factors specifically in the animal through the recognition of other environmental signals encountered inside the host.

Therefore, more studies are needed to determine the molecular mechanisms underlying the regulation of virulence gene expression in response to temperature in these bacteria in order to address rational strategies to deal with bacterial diseases in the aquaculture industry.

## Conflict of Interest Statement

The authors declare that the research was conducted in the absence of any commercial or financial relationships that could be construed as a potential conflict of interest.
